# Clinical characteristics and risk factors analysis of viral shedding time in mildly symptomatic and asymptomatic patients with SARS-CoV-2 Omicron variant infection in Shanghai

**DOI:** 10.3389/fpubh.2022.1073387

**Published:** 2023-01-04

**Authors:** Ran Li, Chen Jin, Liya Zhang, Dehong Kong, Kerong Hu, Miao Xuan, Qi Liu, Shaohui Li, Keqin Zhang, Ying Xue

**Affiliations:** ^1^Department of Endocrinology and Metabolism, Tongji Hospital, School of Medicine, Tongji University, Shanghai, China; ^2^Department of Orthopedics, Tongji Hospital, School of Medicine, Tongji University, Shanghai, China; ^3^Department of Otorhinolaryngology - Head and Neck Surgery, Tongji Hospital, School of Medicine, Tongji University, Shanghai, China

**Keywords:** Omicron, viral shedding time, traditional Chinese medicine, age, hypertension

## Abstract

**Objective:**

To analyze the clinical characteristics and risk factors of viral shedding time in mildly symptomatic and asymptomatic patients with the severe acute respiratory syndrome coronavirus 2 (SARS-CoV-2) Omicron variant (BA.2 and BA2.2) infection in Shanghai, and the effect of traditional Chinese medicine (TCM) treatment, so as to provide a reference basis for epidemic prevention, control and clinical treatment.

**Methods:**

A total of 6,134 asymptomatic or mildly symptomatic Omicron-infected patients admitted to Tianhua Road fangcang shelter hospital in Jinshan, Shanghai, between April 2022 and May 2022 were included. Demographic characteristics and clinical histories were collected and compared in subgroups according to the different durations of viral shedding. Spearman's correlation analysis was performed to explore the association between virus shedding time and clinical variables. Multiple linear regression was used to evaluate the risk factors for viral shedding time.

**Result:**

Most patients with asymptomatic and mildly symptomatic Omicron infection were male, and more than half of patients had a viral shedding time of 8–15 days. The patients were divided into three groups according to the time of viral shedding: short-duration (≤ 7 days), intermediate-duration (8–15 days) and long-duration group (≥16 days). The proportion of patients aged ≤ 29 years was the highest in the short-duration group (30.2%), whereas the proportion of patients aged 50–64 yeas was the highest in the long-duration group (37.9%). The proportion of patients with the chronic non-communicable diseases among the short-, intermediate- and long-duration groups was 6.2, 9.4, and 14.9%, respectively. Among them, hypertension was the most found (4.9, 7.8, and 11.7%, respectively). By multivariate analyses, we identified that viral shedding time of Omicron variants was independently negatively correlated with male patients, TCM treatment, and manual laborers, while it was independently positively associated with age and hypertension. Additionally, TCM treatment could significantly shorten the length of viral shedding time, especially for men, age ≥30 years, comorbid chronic non-communicable diseases, unemployed people and manual worker.

**Conclusions:**

Our results suggested that age and hypertension were independent risk factors for the duration of viral shedding in asymptomatic and mildly symptomatic omicron infected patients. TCM can effectively shorten viral shedding time.

## Introduction

Since the outbreak of the coronavirus disease 2019 (COVID-19) pandemic, there have been several variants of the severe acute respiratory syndrome coronavirus 2 (SARS-CoV-2), one of which is the Omicron variant (1–5). The Omicron variant was first identified in South Africa and Botswana, and reported to the World Health Organization on November 24, 2021 as a novel variant. It has replaced the Delta strain as the major prevalent strain worldwide, and its high transmissibility and immune evasion capabilities have attracted global concerns (5–9).

In early March 2022, a major outbreak of SARS-CoV-2 Omicron variant spread rapidly throughout Shanghai, China. This SARS-CoV-2 epidemic was the Omicron subtype BA.2 and BA.2.2. To effectively implement the “dynamic zeroing” policy, China established a large number of fangcang shelter hospitals to manage asymptomatic and mildly symptomatic patients infected with Omicron BA.2 and BA2.2 (10, 11). Recently, a report from Chen et al. (12) systematically described the epidemiological features and transmission dynamics of the Omicron epidemic in Shanghai, and revealed that from February 26 to June 30, 2022, the overall infection rate, severe/critical infection rate and mortality rate were 2.74 (95% CI: 2.73 to 2.74) per 100 individuals, 6.34 (95% CI: 6.02–6.66) per 100,000 individuals and 2.42 (95% CI: 2.23–2.62) per 100,000 individuals, respectively. The rate of severe/critical infection and mortality increased significantly with age, with the highest rates among those aged 80 years or older, where the rate of serious/critical infection was 125.29 (95% CI: 117.05–133.44) per 100,000 and the mortality rate was 57.17 (95% CI: 51.63–62.71) per 100,000 individuals, respectively. At that time, the total number of reported infections was 626,811, including 568,811 (90.75%) asymptomatic infections and 58,000 (9.25%) symptomatic cases (12).

A key infected population in this outbreak was the asymptomatic and mildly symptomatic patients, as Omicron tended to infect primarily the upper respiratory tract, not the lungs (13, 14). The insidious symptoms of these patients can easily lead to underdiagnosis, resulting in the continued spread of Omicron in society and making the epidemic difficult to prevent and control (15), which may be one of the main factors in the rapid global spread of Omicron (4, 16). Currently, isolation measures for Omicron-infected individuals and close contacts are used in several countries to cut off the transmission route (4). Inappropriate and excessively prolonged quarantine may place a huge burden on society and affect the psychological well-being of Omicron-infected individuals, whereas too short an isolation period may lead to the spread of the virus. Therefore, assessing the viral shedding time of SARS-CoV-2 and thus determining the optimal duration of isolation is of great practical importance (17). In addition, accumulating evidence has shown that traditional Chinese medicine (TCM) played an important role in alleviating patients' symptoms, shortening the duration of illness, delaying disease progression and reducing mortality (18, 19).

Given the serious public health threat posed by Omicron variants and the potential to undermine global efforts to control the COVID-19 pandemic, in-depth research and comprehensive understanding of Omicron is urgently needed. However, information on the characterization of the duration of nucleic acid turning negative (i.e., virus shedding) in the Omicron outbreak in Shanghai remains limited. Therefore, we aimed to provide a detailed description of the clinical characteristics of asymptomatic and mildly symptomatic patients with SARS-CoV-2 Omicron variants infection (both BA.2 and BA.2.2) during the epidemic period in Shanghai, as well as to analyze the risk factors associated with the timing of viral shedding. In addition, we investigated the efficacy of TCM in order to develop more effective treatment strategies in the future.

## Methods

### Study design and population

The study was approved by the Ethics Committee of Tongji Hospital, Tongji University School of Medicine (K-W-2022-010). In this retrospective study, we included a total of 6,134 asymptomatic or mildly symptomatic patients admitted to Tianhua Road fangcang shelter hospital in Jinshan, Shanghai, between April 2022 and May 2022.

The definitions of confirmed COVID-19 cases and SARS-CoV-2 infections were based on the Clinical Guidance for the Diagnosis and Treatment of COVID-19 Pneumonia (Trial ninth edition) published by the National Health Commission of China (20). SARS-CoV-2 infections, including asymptomatic infections and symptomatic cases, were ascertained by Reverse Transcription-Polymerase Chain Reaction (RT-PCR). Asymptomatic infections were defined as RT-PCR-confirmed individuals who (1) did not meet any of the following clinical criteria: fever, cough, sore throat, and other self-perceived and clinically identifiable symptoms or signs; and (2) had no radiographic evidence of pneumonia. Symptomatic cases were further categorized by clinical severity as mild, moderate, severe and critical cases. Mild cases were defined as those with mild symptoms such as fever, fatigue, loss of taste/smell, but without radiographic evidence of pneumonia. Moreover, the population was screened for self-administered rapid antigen testing as a complement to nucleic acid testing; any positive antigen test results required nucleic acid testing for confirmation. Routine surveillance was based primarily on symptom monitoring in medical institutions. Patients without a consent form or complete medical history were excluded from this study.

Participants received an oropharyngeal swab for SARS-CoV-2 RT-PCR test once daily during hospitalization, and were considered to be virus cleared when two consecutive SARSCoV-2 RT-PCR tests for nucleic acid were negative [cycle thresholds >35 for both the nucleocapsid protein (N) gene and open reading frame (ORF) 1ab gene], tested at intervals of at least 24 h. The duration of viral shedding was defined as the first day of positive nucleic acid test to the date of the first negative test of consecutive negative results.

### Data sources and collection

Baseline information (i.e., sex, age and ethnicity), clinical manifestations, comorbidities (i.e., hypertension, type 2 diabetes mellitus and cardiac-cerebral vascular disease), TCM treatment or not, type of occupation, vaccination status, and nucleic acid test results were recorder. The kinds of TCM treatment include, Jinhua Qinggan Granule, Lianhua Qingwen Capsule, Shufeng Jiedu Capsule, Huoxiang Zhengqi Capsule, Jingyin gubiao Tang, Longyi Fangyi Tang, Longfei zhengqi Tang and Maxing Zhiliao Tang. The main source of information was medical records of the patients (both electronic and paper).

### Statistical analyses

All statistical analyses were performed using SPSS software (version 19.0, SPSS Inc). Duration of viral shedding was a continuous variable, conforming to a normal distribution, and expressed as mean ± standard deviation. Independent samples *t-*test was used for comparison between two groups. Categorical data were expressed as number of cases (percentage), and compared using Pearson's chi-Square test. Spearman's correlation analysis was performed to explore the association between virus shedding time and clinical variables. We evaluated factors affecting viral clearance using multiple linear regression analysis. Covariates included sex, age, different comorbidities, vaccination status, TCM treatment, and type of occupation. *P* < 0.05 was considered to be statistically significant.

## Results

### Clinical manifestations of enrolled patients

**Figure 1:**
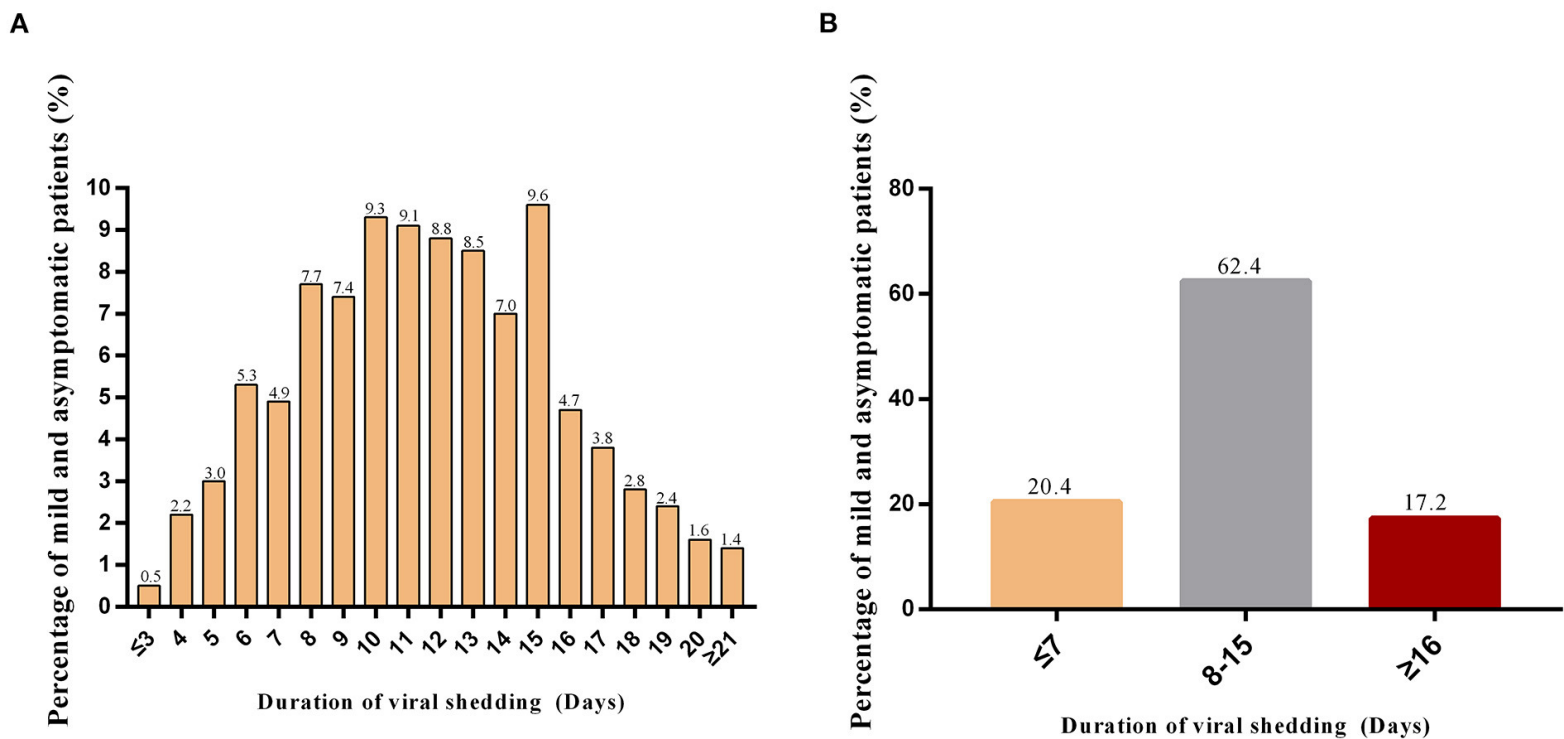
Distribution of viral shedding time* for asymptomatic and mildly symptomatic Omicron-infected patients. **(A)** Distribution of viral shedding times; **(B)** The proportion of patients in the three groups with different viral shedding times. *The duration of viral shedding was defined as the first day of a positive nucleic acid test to the date of the first negative test of consecutive negative results. Participants received an oropharyngeal swab for SARS-CoV-2 RT-PCR test once daily during hospitalization, and were considered virus cleared when two consecutive SARS-CoV-2 RT-PCR tests were negative for nucleic acid [cycle threshold >35 for both nucleocapsid protein (N) gene and open reading frame (ORF) 1ab gene], tested at intervals of at least 24 h.

A total of 6,134 asymptomatic and mildly symptomatic patients were included in the present study. Figure 1 showed the distribution of viral shedding times for the SARS-CoV-2 Omicron variant. The results revealed that more than half of the patients had a viral shedding time of 8–15 days (Figure 1A). Therefore, we divided all enrolled patients into three groups according to the duration of viral shedding: short-duration group (≤ 7 days, 1,249 patients, 20.4%), intermediate-duration group (8–15 days, 3,832 patients, 62.4%) and long-duration group (≥16 days, 1,059 patients, 17.2%) (Figure 1B). The basic characteristics of the three groups were listed in Table 1. In all three groups, the number of Omicron infections was obviously higher in males than in females. The mean age of the participants was 42 years (range 2 to 76 years old). Within the three groups, patients were further divided by age into 5 subgroups: ≤ 29, 30–39, 40–49, 50–64, and ≥65 years. In the short-duration group, the proportion of patients aged ≤ 29 years was the highest among the five age subgroups (30.2%), while for the intermediate-duration group and the long-duration group, the highest proportion was aged 50–64 years (29.2% and 37.9%, respectively). Moreover, the majority of patients in the three groups were Han Chinese (97.1, 97.5, and 98.2%, respectively). With regard to chronic non-communicable diseases, hypertension was the most found, followed by type 2 diabetes mellitus, and cardiac-cerebral vascular disease. Moreover, the longer the duration of the disease, the higher the proportion of comorbidities. For example, in the short-duration group, the proportion of comorbid chronic non-communicable diseases was only 6.2%, however, this proportion increased to 14.9% in the long-duration group.

**Table 1:** The basic characteristics of enrolled patients.

	**Short-duration** **(≤ 7 days)**	**Intermediate-duration** **(8-15 days)**	**Long-duration** **(≥16 days)**	***P*-value**
**Sex**				< 0.001
Female	353 (28.3%)	1,083 (28.3%)	421 (39.8%)^*^^#^	
Male	896 (71.7%)	2,743 (71.7%) ^†^	638 (60.2%)^*^^#^	
**Age, years**				< 0.001
≤ 29	377 (30.2%)	827 (21.6%)^*^	160 (15.1%) ^#^	
30–39	343 (27.5%)	934 (24.4%)	187 (17.7%)^*^^#^	
40–49	221 (17.7%)	841 (22.0%)^*^	251 (23.7%)^*^	
50–64	285 (22.8%)	1,119 (29.2%)^*^	401 (37.9%)^*^^#^	
≥65	23 (1.8%)	105 (2.7%)	60 (5.7%)^*^^#^	
**Race**				0.232
Han	1,213 (97.1%)	3,730 (97.5%)	1,040 (98.2%)	
other	36 (2.9%)	96 (2.5%)	19 (1.8%)	
**Chronic non-communicable diseases**	78 (6.2%)	360 (9.4%)^*^	148 (14.9%)^*^^#^	< 0.001
Hypertension	61 (4.9%)	300 (7.8%)^*^	124 (11.7%)^*^^#^	< 0.001
Type 2 diabetes mellitus	17(1.4%)	86 (2.2%)	30 (2.8%)^*^	0.046
Cardiac-cerebral vascular disease	13 (1%)	18 (0.5%)	17 (1.6%) ^#^	0.001
**TCM**	274 (21.9%)	767 (20.0%)	128 (12.1%)^*^^#^	< 0.001
**Occupation type**				< 0.001
Worker with no employment	52 (4.2%)	195 (5.1%)	112 (10.6%)^*^^#^	
Mental worker	244 (19.5%)	690 (18.0%)	190 (17.9%)	
Manual worker	953 (76.3%)	2,941 (76.9%)	757 (71.5%)^*^^#^	
**Vaccination status**				0.134
Unvaccinated	254 (20.3%)	702 (18.3%)	233 (22.0%)	
Partially vaccinated	46 (3.7%)	130 (3.4%)	30 (2.8%)	
Fully vaccinated	355 (28.4%)	1,083 (28.3%)	283 (26.7%)	
Fully vaccinated plus booster dose	594 (47.6%)	1,911 (49.9%)	513 (48.4%)	

^*^P < 0.05 vs. short-duration group;

^#^P < 0.05 vs. intermediate-duration group.

TCM, traditional Chinese medicine.

Some of the infected patients received TCM treatment. Among them, 274 (21.9%) infections were treated with TCM in the short-duration group, 767 (20.0%) cases in the intermediate-duration group, and 128 (12.1%) cases in the long-duration group. Different types of occupation were present among asymptomatic and mildly ill patients, with manual workers predominating, followed by mental workers and finally worker with no employment. Interestingly, the type of occupation may also influence the time of viral shedding. Although the proportion of manual workers was higher than that of mental workers and workers with no employment within the three viral shedding time groups, however, the proportion of manual workers decreased in the long-duration group (71.5%) compared to the short-duration group (76.3%), while the proportion of jobless people increased in the long-duration group (10.6%) compared to the short-duration group (4.2%).

In addition, the largest proportion of patients in all three groups had received a full vaccination plus booster (47.6, 49.9, and 48.4%, respectively), followed by those who had received a full vaccination (28.4, 28.3, and 26.7%, respectively). Although the proportion of vaccination status was not statistically different between the three groups, numerically the highest proportion of unvaccinated in the long-duration group (22%), which was higher than the short-duration group (20.3%) and the intermediate group (18.3%). Mildly symptomatic and asymptomatic patients had received COVID-19 vaccine produced by five Chinese companies, including Sinovac Life Science (CoronaVac), Sinopharm Beijing Bio-Institute of Biological Products (BBIBP), Sinopharm Wuhan Institute of Biological Products (WIBP), CanSinoBio and Zhifei Longcom. Of all three groups, the largest proportion of patients received the CoronaVac vaccine (53.6, 55.4, and 51.5%, respectively), followed by those who received the BBIBP vaccine (18.3, 19.0, and 19.8%, respectively) (Supplementary Figure 1).

### Risk factors for time to viral shedding of Omicron variants in asymptomatic and mildly symptomatic patients

We included 6,134 patients with viral shedding time into analysis. Spearman's correlation analysis suggested that the duration of viral shedding in asymptomatic and mildly infected patients with Omicron was negatively correlated with sex, TCM therapy and occupation type, and positively correlated with age, hypertension, type 2 diabetes mellitus and vaccination status (Table 2).

**Table 2:** Spearman's correlation analysis of viral shedding time and clinical characteristics.

	**Duration of viral shedding**	**Sex**	**Age**	**Race**	**Hypertension**	**Type 2 diabetes mellitus**	**Cardiac-cerebral vascular disease**	**TCM**	**Occupation type**	**Vaccination status**
Duration of viral shedding	1									
Sex	−0.103^**^	1								
Age	0.170^**^	−0.011	1							
Race	0.020	0.005	0.098^**^	1						
Hypertension	0.090^**^	−0.012	0.266^**^	0.031^*^	1					
Type 2 diabetes mellitus	0.044^*^	−0.002	0.125^**^	0.009	0.214^**^	1				
Cardiac-cerebral vascular disease	0.016	0.014	0.046^**^	−0.010	0.084^**^	0.025	1			
TCM	−0.096^**^	0.076^**^	0.086^**^	−0.014	0.013	−0.010	0.013	1		
Occupation type	−0.053^**^	−0.158^**^	0.101^**^	−0.007	−0.028^*^	−0.070	−0.024	0.063^**^	1	
Vaccination status	0.013	0.005	0.122^**^	0.016	0.009	−0.013	−0.013	0.006	0.083^**^	1

^*^P < 0.05,

^**^P < 0.01.

TCM, traditional Chinese medicine.

The univariate analyses showed male patients, patients treated with TCM, mental workers, and manual workers were negatively association with viral shedding time of the Omicron variant, while age ≥30 years, and combined with hypertension and type 2 diabetes mellitus were positively correlated to the duration of virus clearance (Table 3). By multivariate analysis, we identified male patients, TCM-treated patients, and manual workers were independently and negatively associated with viral shedding periods of the Omicron variant [male: β, −0.764 (95%CI: −1.023 to −0.505); *P* < 0.001. TCM therapy: β, −1.167 (95%CI: −1.466 to −0.868); *P* < 0.001. Manual worker: β, −0.753 (95%CI: −1.293 to −0.213); *P* = 0.006]. More importantly, we found an independent positive association between age, hypertension and the duration of viral shedding: age 30–39 years [β, 0.403 (95%CI: 0.058–0.748); *P* = 0.022], 40–49 years [β, 1.689 (95%CI: 1.326–2.052); *P* < 0.001], 50–64 years [β, 1.718 (95%CI: 1.371–2.065); *P* < 0.001], ≥ 65 years [β, 2.389 (95%CI: 1.622–3.157); *P* < 0.001], hypertension [β, 0.872 (95%CI: 0.414–1.330); *P* < 0.001] (Table 3).

**Table 3:** Risk factors of virus clearance time among asymptomatic and mildly symptomatic patients.

	**Univariate analysis**			**Multivariate analysis**		
	β	**95%CI**	***P***	β^a^	**95%CI**	**P**
**Sex**						
female	Ref	-	-	Ref	-	-
male	−0.982	−1.241 to −0.723	< 0.001	−0.764	−1.023 to −0.505	< 0.001
**Age, years**						
≤ 29	Ref	-	-	Ref	-	-
30–39	0.354	0.006 to 0.702	0.046	0.403	0.058–0.748	0.022
40–49	1.574	1.217 to 1.931	< 0.001	1.689	1.326 to 2.052	< 0.001
50–64	1.644	1.313 to 1.976	< 0.001	1.718	1.371 to 2.065	< 0.001
≥65	2.807	2.088 to 3.526	< 0.001	2.389	1.622 to 3.157	< 0.001
**Race**						
Other	Ref	-	-	-	-	-
Han	0.744	−0.027 to 1.516	0.059	-	-	-
Comorbidities	1.467	1.062 to 1.872	< 0.001	-	-	-
Hypertension	1.581	1.140 to 2.022	< 0.001	0.872	0.414 to 1.330	< 0.001
Type 2 diabetes mellitus	1.061	0.240 to 1.882	0.011	−0.067	−0.894 to 0.759	0.873
Cardiac-cerebral vascular disease	1.027	−0.330 to 2.384	0.138	-	-	-
TCM	−1.109	−1.413 to −0.806	< 0.001	−1.167	−1.466 to −0.868	< 0.001
**Occupation type**						
Worker with no employment	Ref	-	-	Ref	-	-
Mental worker	−1.455	−2.021 to −0.899	< 0.001	−0.146	−0.746 to 0.455	0.635
Manual worker	−1.657	−2.168 to −1.145	< 0.001	−0.753	−1.293 to −0.213	0.006

^a^adjusted sex, age, comorbidities, traditional Chinese medicinal, occupation type.

-, data not applicable.

Ref, reference; CI, confidence interval; TCM, traditional Chinese medicine.

### Effect of traditional Chinese medicinal (TCM) therapy on the duration of viral shedding in different populations

To investigate the effect of TCM therapy on the duration of viral shedding in asymptomatic and mildly symptomatic patients with Omicron infection, we classified patients into non-treatment, and TCM treatment groups according to whether they received TCM therapy or not. As shown in Table 4, the duration of viral clearance was significantly shorter in patients who received TCM treatment compared to the non-treatment group (11.10 ± 4.360 days vs. 12.21 ± 4.847 days, *P* < 0.001). More importantly, the duration of viral shedding was obviously shorter in asymptomatic and mildly symptomatic Omicron infected patients who were male, age ≥30 years, accompanied by chronic non-communicable diseases, workers with no employment and manual workers in the treatment group compared to the non-treatment group (*P* < 0.05). Notably, as the age of the infected patients increased, the TCM-treated group showed a more pronounced reduction in viral clearance time compared to the untreated group.

**Table 4:** Effect of treatment with TCM therapy on duration of viral shedding.

	**Number of cases** **(non-treatment/** **treatment)**	**Non-treatment** **(days)**	**Treatment** **(days)**	Treatment-non-treatment^*^ **(days)**	***P*-value**
	4,965/1,169	12.21 ± 4.847	11.10 ± 4.360	1.11	< 0.001
**Sex**					
Female	1,587/270	12.73 ± 4.978	12.43 ± 4.449	0.3	0.351
Male	3,378/899	11.97 ± 4.766	10.70 ± 4.255	1.27	< 0.001
**Age**					
≤ 29	1,158/206	11.09 ± 4.791	10.52 ± 4.150	0.57	0.107
30–39	1,229/235	11.51 ± 4.359	10.61 ± 4.891	0.9	0.005
40–49	1,036/277	12.94 ± 4.961	11.26 ± 4.089	1.68	< 0.001
50–64	1,395/410	13.00 ± 4.879	11.48 ± 4.261	1.52	< 0.001
≥65	147/41	14.31 ± 5.225	12.04 ± 4.485	2.27	0.012
Chronic non-communicable diseases	464/122	13.80 ± 5.406	11.53 ± 4.030	2.27	< 0.001
Hypertension	384/101	13.90 ± 5.586	11.77 ± 3.789	2.2	< 0.001
Type 2 diabetes mellitus	111/22	13.39 ± 4.074	11.26 ± 4.659	2.13	0.031
Cardiac-cerebral vascular disease	36/12	14.02 ± 5.576	10.00 ± 5.519	4.02	0.035
**Occupation**					
Worker with no employment	302/57	13.81 ± 5.126	12.02 ± 4.626	1.79	0.015
Mental worker	965/159	12.19 ± 5.154	11.33 ± 4.730	0.86	0.051
Manual worker	3,698/953	12.09 ± 4.718	11.01 ± 4.276	1.08	< 0.001

Data are expressed as mean ± SD.

^*^The difference of the viral shedding time before and after TCM treatment, that is, the absolute value of the mean of the Treatment group minus the mean of the Non-treatment group.

TCM, traditional Chinese medicine.

## Discussion

This study provides a comprehensive analysis of the characteristics of the timing of viral shedding and associated risk factors in asymptomatic and mildly symptomatic patients infected with the Omicron variant of SARS-CoV-2. Our findings revealed an independent negative correlation between the duration of viral shedding and men, TCM treatment and manual workers, and an independent positive correlation with age and hypertension. Furthermore, TCM treatment significantly reduced the time to viral shedding in asymptomatic and mildly symptomatic patients with Omicron infection, especially in male, aged ≥30 years, with chronic non-communicable diseases, workers with no employment and manual workers. These findings may help health professionals to better understand the characteristics of the Omicron variant and to adjust treatment regimens.

Our study found a higher proportion of males than females were infected with Omicron variant in mildly symptomatic and asymptomatic patients, and men was independently and negatively correlated with the duration of viral shedding. In addition, the duration of viral clearance increased with age, and age was independently and positively correlated with time to virus shedding. We speculate that these differences may be due to the differential effects of age and sex on the immune system. Using single-cell transcriptome sequencing, one study has found that females have more plasma cells in the circulation and a stronger B-cell-activating factor of the tumor necrosis factor family (BAFF)/proliferation-inducing ligand (APRIL) system, which is consistent with a stronger adaptive immune response. In contrast, males have a higher percentage of natural killer (NK) cells in their blood and higher expression of certain pro-inflammatory genes (21). During the COVID-19 pandemic in China, men had higher hospital admission and mortality rates than women (22). Besides, the progressive degeneration of the immune system and the persistence of a chronic inflammatory state in the elderly lead to increased susceptibility to infection and reduced ability to recover (23). Previous clinical studies identified that age was positively associated with disease severity and mortality after SARS-CoV-2 infection in the COVID-19 pandemic and was a strong risk factor (23). A basic study demonstrated that, compared to young mice, aging mice had significantly diminished interferon and adaptive antibody responses to SARS-CoV-2 infection (24). However, the specific mechanisms underlying the age and sex differences in Omicron infection need to be further studied in the future. Interestingly, the type of occupation also influenced the duration of viral shedding. Manual workers, who accounted for the majority of asymptomatic and mildly symptomatic infections, were independently and negatively associated with the duration of viral shedding. This may be related to the fact that manual workers are predominantly males and that the wide range of activities of manual workers leads to a greater chance of being infected by Omicron.

Multiple studies have established that patients with comorbidities such as hypertension, diabetes and cardiac-cerebral vascular disease are more susceptible to SARS-CoV-2 (25, 26). One study included 1099 patients diagnosed with COVID-19, 173 of whom were severely ill with comorbidities of hypertension (23.7%), diabetes mellitus (16.2%), coronary heart diseases (5.8%), and cerebrovascular disease (2.3%) (27). In another study, of the 140 patients who were admitted to hospital for COVID-19, 30% had hypertension and 12% had diabetes (28). A pooled analysis demonstrated hypertension increased the risk of exacerbation and death of COVID-19 by up to 2.5-fold, particularly in the elderly (29). This may be due to the fact that overexpression of the angiotensin-converting enzyme 2 (ACE-2) receptors in hypertensive patients is more likely to mediates viral infections in the lungs, leading to more severe outcome than other clinical conditions (25). Additionally, the use of drugs such as ACE-2 inhibitors and angiotensin receptor blockers (ARBs) in patients with hypertension and cardiovascular disease leads to ACE-2 overexpression *in vivo*, thereby increasing the risk of SARS-CoV-2 infection (25). Furthermore, elevated ACE-2 expression and cytokines storm in diabetic patients also increase the risk of SARS-CoV-2 infection (25). In the present study, asymptomatic and mildly symptomatic patients with Omicron infection accompanied by chronic non-communicable diseases such as hypertension, type 2 diabetes mellitus and cardiac-cerebral vascular disease had a significantly longer time to viral shedding compared to those without chronic non-communicable disease. However, only hypertension was independently and positively associated with time to viral shedding.

Approximately 19.1% (1,169/6,134) of the asymptomatic and mildly symptomatic patients in this study received TCM treatment. The results showed that the time to viral shedding was significantly shorter in patients who received TCM treatment compared to those without TCM treatment, and there was an independent negative correlation between TCM treatment and time to viral shedding. Notably, as the age of the infected patients increased, the reduction in time to viral shedding was more pronounced in TCM-treated than untreated patients. These demonstrate the effectiveness and importance of TCM treatment for mildly symptomatic and asymptomatic patients infected with Omicron. Clinical evidence has previously shown that TCM can significantly alleviate the clinical symptoms, effectively reduce the incidence of severe illness and decrease all-cause mortality in patients with COVID-19 (18). The mechanism of TCM treatment for COVID-19 may be to prevent SARS-CoV-2 from entering cellular host by reducing viral replication and transcription, interfering with viral binding to ACE-2 receptors, and decreasing the expression of type 2 transmembrane serine protease (TMPRSS2) (18). Besides, TCM also regulates interleukin-6 (IL-6), a cytokine storm mediator, to improve COVID-19 symptoms (18, 19).

Several limitations should be mentioned in our study. Firstly, we only included patients from one fangcang shelter hospital in Shanghai, which is not fully representative of the overall population of Omicron-infected patients. Secondly, due to the retrospective design of this study and the lack of relevant laboratory tests (such as blood indicators), the analysis of risk factors for time to viral shedding may not be comprehensive. Thirdly, the choice of TCM therapies is mainly based on the patient's wish to use the medicine and the experience of the clinician who sees them. As a result, there may be artificial bias in the choice of TCM treatment. Finally, as only mildly symptomatic and asymptomatic infected patients were admitted to this shelter hospital, we were unable to analyze the time to viral shedding and associated risk factors in moderate, severe and critical cases.

## Conclusions

Our study describes in detail the clinical characteristics of the period of viral shedding and associated risk factors in mildly symptomatic and asymptomatic patients infected with SARS-CoV-2 Omicron variant (including BA.2 and BA.2.2). We found that the duration of viral shedding was 8–15 days in most patients. Age and hypertension were positively associated with time to viral shedding and were independent risk factors for it. However, males and manual workers were independently and negatively related to the duration of viral shedding. Additionally, TCM treatment was effective in shortening the duration of viral shedding time, especially in males, ≥30 years of age, with chronic non-communicable diseases, workers with no employment and manual workers. This study provides a theoretical basis for clinicians to more rationally determine the time to isolation and provide effective treatment for mildly symptomatic and asymptomatic patients with Omicron infection.

## Data availability statement

The original contributions presented in the study are included in the article/Supplementary material, further inquiries can be directed to the corresponding author/s.

## Ethics statement

The studies involving human participants were reviewed and approved by the Ethics Committee of Tongji Hospital, Tongji University School of Medicine (K-W-2022-010). Written informed consent to participate in this study was provided by the participants' legal guardian/next of kin.

## Author contributions

RL, CJ, and LZ analyzed the patient data and drafted the manuscript. DK and KH contributed to data interpretation. MX coordinated the research. QL and SL contributed to data interpretation and critical revision of the manuscript. YX and KZ designed the study, revised, and prepared the final version of the manuscript. All authors read and approved the final manuscript.
