# Supplementary material for: Clinical characteristics and risk factors analysis of viral shedding time in mildly symptomatic and asymptomatic patients with SARS-CoV-2 Omicron variant infection in Shanghai
Source: Front Public Health. 2023 Jan 4;10:1073387. doi: 10.3389/fpubh.2022.1073387 (PMC9845758; doi:10.3389/fpubh.2022.1073387)
Supplement: Supplementary file 1 [file Data_Sheet_1.PDF]

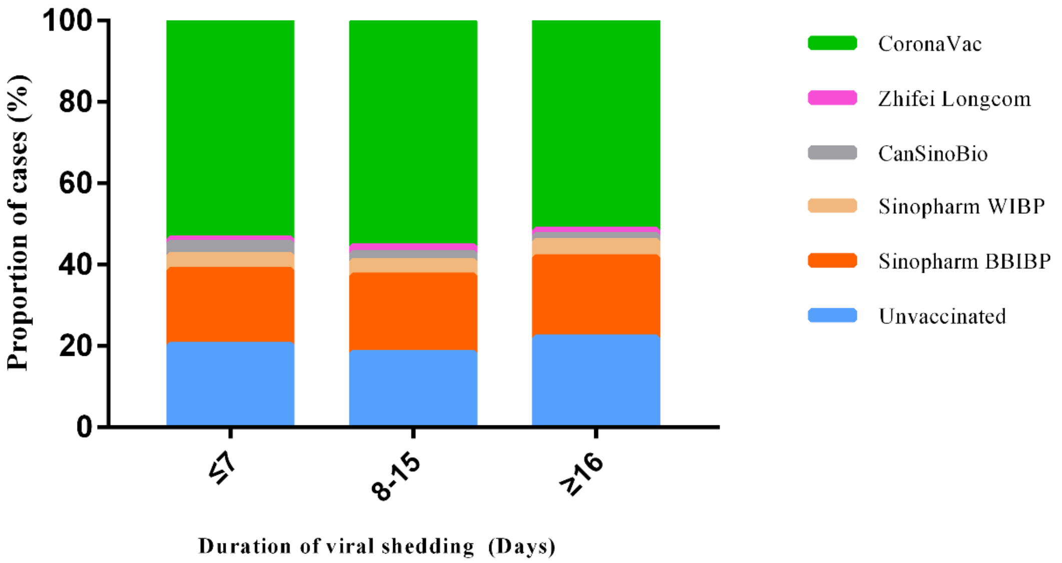


**Supplementary Figure 1.** The proportional distribution of vaccine types administered to infected patients in three different virus shedding duration groups.

Abbreviations: BBIBP, Beijing Bio-Institute of Biological Products; WIBP, Wuhan Institute of Biological Products.
